# Environmental risk evaluation of overseas mining investment based on game theory and an extension matter element model

**DOI:** 10.1038/s41598-021-95910-x

**Published:** 2021-08-11

**Authors:** Hujun He, Rui Xing, Ke Han, Junjie Yang

**Affiliations:** 1 School of Earth Science and Resources, Chang’an University, Xi’an, 710054 China; 2 Key Laboratory of Western Mineral Resources and Geological Engineering, Ministry of Education, Xi’an , 710054 China

**Keywords:** Ecology, Environmental sciences, Environmental social sciences

## Abstract

Taking into account the limitations of the single weighting method presently used for the environmental risk evaluation of overseas mining investment, an improved extension evaluation method based on game theory was developed. The method was then applied to real data from the Philippines and used to establish the congener element object and classical domain of the environmental risk of mining investment in the Philippines, based on extension matter element theory. The optimal index weights, based on a balance of subjective and objective results, were obtained from game theory, the analytic hierarchy process, and entropy weight theory. This enabled calculation of the association function values of evaluation indexes in the Philippines and the environmental risk level of overseas mining investment. Finally, given the weighting and association function values, the environmental risk level of mining investment in the Philippines was determined to be level II (higher risk). These results show that the proposed model is effective for evaluating the environmental risk of overseas mining investment.

## Introduction

**Figure 1:**
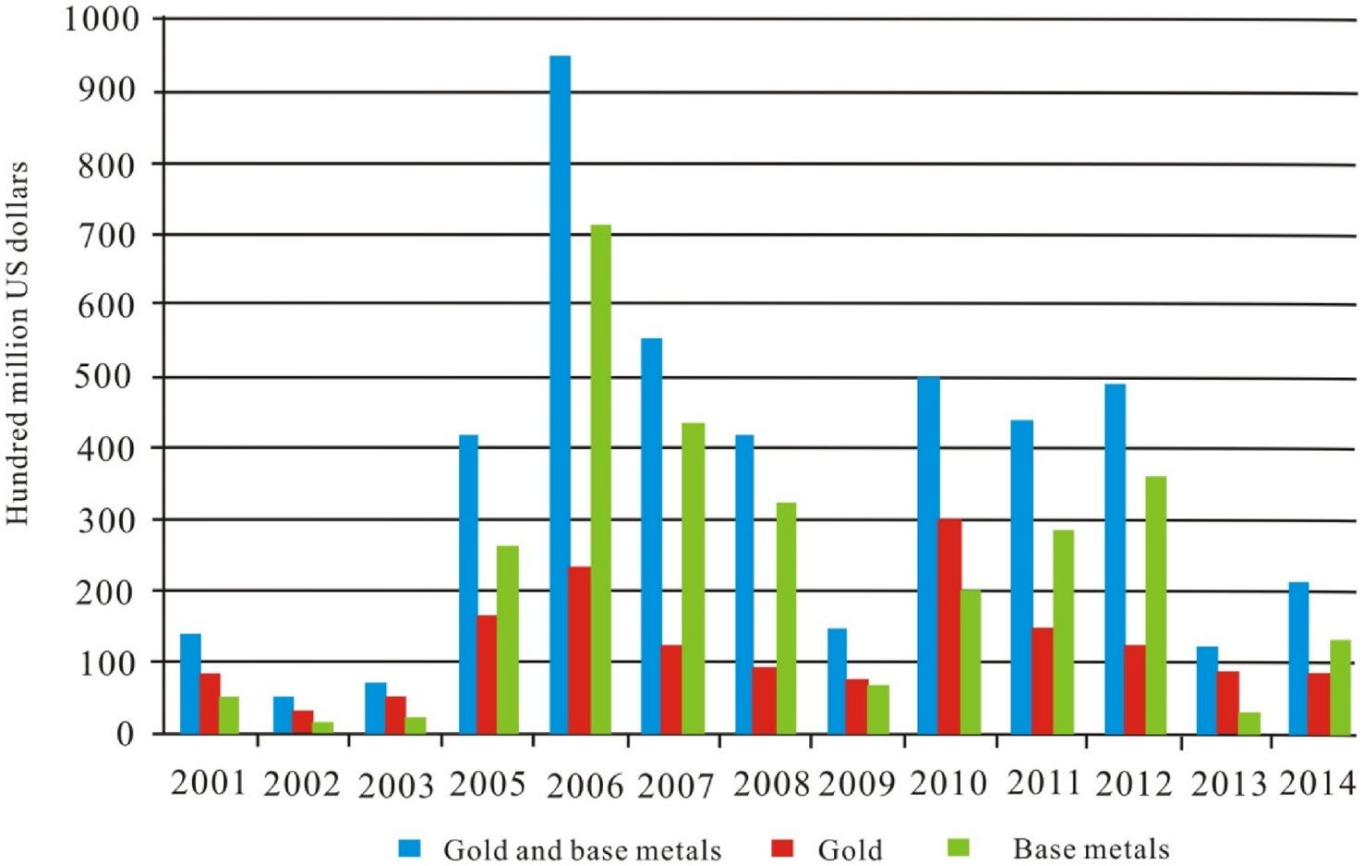
Trends of global mining investment for China’s mining companies in the past 10 years ( Source: SNL Association).

Mineral resources are necessary for national development, and sustainable mining is important for improving economic efficiency and enhancing industrial production and economic growth. China has become the world’s largest consumer of mineral resources, and resource and energy shortages represent serious challenges to China’s economic stability and sustainable development. To compensate for the shortage of mineral resources in China, overseas mining investment is being explored. Mining enterprises in China have responded positively to the strategic vision of the state in developing the Silk Road Economic Belt and 21^st^ Century Maritime Silk Road. Mineral resource exploration and development overseas are also accelerating (Fig. 1). However, overseas mining investment is an economic activity requiring a large amount of investment, a long development period, and ability to sustain high risk^1–5^. There are many uncertain and uncontrollable risk factors that pose challenges to overseas mining investment. Therefore, objective risk evaluation of the investment environment of the host country is important to mining companies^6–12^.

The gradual establishment of global free trade and open economies has led to studies of risk evaluation of overseas mining investment. At present, the main methods for evaluating the risk of overseas mining investment include analytic hierarchy process (AHP)^13,14^, data envelopment analysis (DEA)^15^, artificial neural network (ANN) analysis^16^, fuzzy comprehensive evaluation method^17^, gray evaluation method^18^, sensitivity analysis^19^, entropy method^20,21^, the Planned Economy Country Risk Model (PERM)^22^, rough set (RS) ^23^, value at risk (VaR)^24^, full probability analysis^25^, variable weight theory^8^, and risk compensation^26^. However, these methods have limitations. For example, in the fuzzy comprehensive evaluation method, a linear weighted-average model is generally used to obtain the evaluation set, and the evaluation results are prone to distortion, failure, homogenization, and jumping. As such, the evaluation process is complex. Given that the grey evaluation method reflects the uncertainty of the overseas mining investment system, it has the advantage of being simple but is limited by low resolution. ANN analysis has some characteristics similar to human evaluation and has the advantage of being rapid and objective. However, for samples with poor coordination, the evaluation results are prone to homogenization^27^. Notably, expert consultation and the AHP and entropy method are the most commonly used methods for evaluating index weights. However, these various weighting methods have their own shortcomings. For example, the expert consultation method and AHP are influenced by the subjective experience of experts and a lack of accuracy. Although the evaluation results are more accurate than the subjective weighting obtained by the entropy method, the importance of expert experience is ignored.

The risk evaluation of overseas mining investment is influenced by many factors, and each factor is restricted and related to each other^6–8^. It is difficult to obtain the specific value of the risk of overseas mining investment under the changing conditions of each factor. To solve these contradictory and incompatible problems, extension matter element theory studies the laws and methods to solve the problem of contradiction from both qualitative and quantitative aspects by the transformation and calculation of the matter element; therefore, it is very relevant to evaluate the nonlinear, time-varying and uncertainty factors influencing the risk of overseas mining investments^27–30^. The index weight is a difficult problem in the application of the extension matter element theory for the risk analysis of overseas mining investment, and it directly affects the accuracy of the final judgement. Therefore, how to overcome the difficulty of determining the weights is an important condition to improve the risk analysis level of overseas mining investment. However, the combination weighting model based on game theory can comprehensively consider the relationship between the indexes, balance the subjective and objective weights, and optimise the index weight values^31,32^.

This paper uses matter element theory to model the risk level, evaluation indexes, and characteristic value of overseas mining investment. This enables determination of the classical domain, joint domain, weighting coefficients, and degree of correlation of the model. The index weighting used here is an improved extension evaluation model based on game theory combination weighting that was developed in this research. We also use our model to evaluate the risk of mining investment in the Philippines and show that it is suitable for overseas mining investment risk evaluation.

The remainder of this paper is organized as follows. “Materials and methods” section describes the data source, the determination method of index weights based on game theory, AHP, entropy weight theory and extension matter element theory. “Result analysis and discussion” section establishes the matter element model of overseas mining investment risk evaluation, analyses the significance of the model and discusses the applicability of the model through the risk evaluation of mining investment in the Philippines. Conclusions are summarised in “Conclusions” section.

## Materials and methods

### Data sources

The data come from the Ministry of Commerce of the People's Republic of China's 2019 Guide to Foreign Investment and Cooperation Country, as well as the websites and research literature from the Fraser Institute and the World Bank. The datasets include 14 factors that influence the environmental risk of overseas mining investment in the Philippines are summarized in Tables 1 and 2. The specific reasons that we choose these data in the Philippines are as follows:

**Table 1:** Evaluation factors and grading standards of environmental risk for an evaluation of overseas mining investment in the Philippines.

_Evaluation index_		_Risk grade_				
		_High risk(_$$ {C_1}$$_)_	_Higher risk(_$$ {C_2}$$_)_	_General risk(_$$ {C_3}$$_)_	_Lower risk(_$$ {C_4}$$_)_	_Low risk(_$$ {C_5}$$_)_
Political policy risk	Political stability	_9.6–12_	_7.2–9.6_	_4.8–7.2_	_2.4–4.8_	_0–2.4_
	Level of government corruption	_0–20_	_20–40_	_40–60_	_60–80_	_80–100_
	Sino-foreign friendship	_>48_	_48–36_	_36–24_	_24–12_	_12–0_
	Mining policy	_0–20_	_20–40_	_40–60_	_60–80_	_80–100_
	Environmental standards	_0–2_	_2–4_	_4–6_	_6–8_	_8–10_
Economic and financial risk	Price level	_>50_	_9–50_	_6–9_	_3–6_	_<3_
	Economic growth rate	_<3_	_3–5_	_5–7_	_7–9_	_>9_
	Exchange rate	_>4.5_	_3–4.5_	_2–3_	_1–2_	_<1_
	Credit rating	_D, C (>4)_	_CCC, CC (3–4)_	_B, BB(2–3)_	_A, BBB(1–2)_	_AA, AAA(0–1)_
Sociocultural risk	Humanistic environment (i.e., community's attitudes, ideas, belief system, cognitive environment, etc.)	_0.1–0.2_	_0.2–0.4_	_0.4–0.6_	_0.6–0.8_	_0.8–1_
	Social security	_8–10_	_6–8_	_4–6_	_2–4_	_0–2_
	Trade union strike	_Many (>4)_	_More (3–4)_	_General (2–3)_	_Fewer (1–2)_	_Few (0–1)_
Infrastructure risk	Information transmission	_0–2_	_2–4_	_4–6_	_6–8_	_8–10_
	Transportation	_10–20_	_20–40_	_40–60_	_50–80_	_>80_

**Table 2:** Risk index data.

_Evaluation index_		Philippine
Political policy risk	Political stability	_7.9_
	Level of government corruption	_34_
	Sino-foreign friendship	_40_
	Mining policy	_38.29_
	Environmental standards	_4_
Economic and financial risk	Price level	_2.69_
	Economic growth rate	_6.58_
	Exchange rate	_0.07_
	Credit rating	_BBB_
Sociocultural risk	Humanistic environment	_0.682_
	Social security	_7.098_
	Trade union strike	_Many_
Infrastructure risk	Information transmission	_4.67_
	Transportation	_76.07_

**Table 3:** Correlation function value of each evaluation index used in an evaluation of overseas mining investment in the Philippines.

_Evaluation index_		Risk grade				
		_High risk_	_Higher risk_	_General risk_	_Lower risk_	_Low risk_
Political policy risk	Political stability	-0.71	-0.3	-0.5	-0.82	-0.9
	Level of government corruption	-0.7	5	-0.5	-0.81	-0.88
	Sino-foreign friendship	-0.67	3	-0.5	-0.8	-0.875
	Mining policy	-0.9145	0.71	-0.5	-0.93	-0.96
	Environmental standards	-1	-1	0	-1	-1
Economic and financial risk	Price level	-0.99	-0.95	-0.91	-0.5	0.69
	Economic growth rate	-0.895	-0.79	-0.58	-0.5	-0.85
	Exchange rate	-0.98	-0.98	-0.965	-0.93	-0.93
	Credit rating	-0.92	-0.86	-0.5	-0.8	-0.8
Sociocultural risk	Humanistic environment	-0.85	-0.77	-0.5	-0.918	-0.059
	Social security	-0.5	-0.098	-0.549	-0.7745	-0.85
	Trade union strike	-1	-1	-1	-1	-1
Infrastructure risk	Information transmission	-0.8	-0.5	-0.33	-0.665	-0.8325
	Transportation	-0.9345	-0.90	-0.8035	2.93	-0.5

The Philippines is a multi-ethnic island nation in Southeast Asia located in the western Pacific Ocean. The country has a total land area of 299,700 km^2^ and a population of 101 million. The Philippines is rich in mineral resources, and the area of known mineralization accounts for 30% of the land area in the country. According to the National Bureau of Geology and Mining in the Philippines, gold, copper, nickel, and chromium reserves rank third, fourth, fifth, and sixth in the world, respectively, in terms of mineral reserves per unit area. Nonferrous metal mining in the Philippines has great potential. To date, 13 types of metal minerals have been discovered, including gold, copper, nickel, aluminium, chromium, silver, lead, and zinc, with total reserves of 7.1 billion tons. Twenty-nine types of nonmetallic minerals have also been discovered with total reserves of 51 billion tons. The Philippines is an important producer and exporter of metallic mineral resources such as copper and nickel^6,33^.

The Philippines has been one of the countries most in favour of overseas mining investment in the region near China. Before the mid-1990s, the Philippines was a favoured country for international mining investors; however, in the late 1990s, changes to national policies and social unrest led to a decline in the mining investment environment. Since January 2003, President Arroyo has proposed a reform of the mining development strategy in the Philippines, and the mining investment environment has improved. However, combined with the political, religious and security issues in the Philippines, especially the peoples’ attitude towards foreign investment, the current mining policy environment in the Philippines is not ideal. Therefore, to comprehensively and objectively understand and analyse the mining investment environment in the Philippines, relevant documents were collated and analysed. Following the principles of importance, practicality, scientificity and systematicness in the design of the index system, the accepted classification rules and data released by authoritative agencies such as the World Bank were used for the evaluation basis^1–8^, which selected 14 factors that have bearing on political policy, economic, financial, sociocultural, and infrastructure risks. The classification standard and valuation of each index are provided in Table 1. According to the classification standard and valuation index objectives, the risks were divided into five levels (i.e., I–V, which reflect high, higher, general, lower, and low risks, respectively). The Philippines’ risk index data are listed in Table 2.

### Determination of index weights: analytical hierarchy process

This method integrates quantitative and qualitative evaluations to improve the accuracy of decision making^32–38^. The basic principles and steps of the AHP method are as follows:

**Step 1:** The complex problem is decomposed to make it multi-element in nature.

**Step 2:** These elements are grouped, and a hierarchical structural model is established.

**Step 3:** A discrimination matrix is constructed, and any two factors are compared with a 1–9 scaling method to obtain the relative importance of each index at each level, which can be expressed quantitatively.

**Step 4:** The largest eigenvalue and the corresponding eigenvector of the discrimination matrix are calculated using the mathematical method, where the eigenvectors and weight coefficient values are listed in terms of the importance of the evaluation factors.

**Step 5:** The consistency of the discrimination matrix is tested based on the consistency index $$ CI$$ calculated as $$CI = \frac{{{\lambda_{\max }} - n}}{n - 1}$$ as well as with the average random consistency index $$RI$$. If the random consistency ratio $$CR = \frac{CI}{{RI}} < 0.10$$, then the results of the hierarchy analysis are considered to be consistent, and the resulting weight distribution values are reasonable. If this is not the case, then the weight coefficient values should be redistributed to adjust the values.

### Entropy weight theory

In information theory, the importance of studying the degree of dispersion of the whole system is central to the entropy method. The specific steps for these calculations are as follows:

**Step 1:** Data collection and sorting: The initial evaluation matrix composed of $m$ evaluation indexes and $n$ evaluation objects is as follows:

1 $$ {{\text{X}}_{{\text{ij}}}} = \left[ {\begin{array}{*{20}{c}} {{x_{11}}}&{{x_{12}}}& \cdots &{{x_{1{\text{n}}}}} \\ {{x_{21}}}&{{x_{22}}}& \cdots &{{x_{2n}}} \\ \vdots & \vdots & \vdots & \vdots \\ {{x_{m1}}}&{{x_{m2}}}& \cdots &{{x_{mn}}} \end{array}} \right] $$

**Step 2:** Data standardization: All index values $$ {x_{ij}}$$ in matrix $$ {X_{ij}}$$ are normalized as follows:

2 $$ {\text{x}}_{ij}^{\prime} = {\raise0.7ex\hbox{${{x_{ij}}}$} \!\mathord{\left/ {\vphantom {{{x_{ij}}} {\sum\limits_{i = 1}^m {{x_{ij}}} }}}\right.\kern-\nulldelimiterspace}\!\lower0.7ex\hbox{${\sum\limits_{i = 1}^m {{x_{ij}}} }$}} $$

**Step 3:** Calculation of information entropy: The entropy of each evaluation index can be obtained from

3 $$ {E_i} = \frac{{\sum\limits_{j = 1}^n {x_{ij}^{\prime}\ln x_{ij}^{\prime}} }}{\ln n} $$

**Step 4:** Calculation weight: The weight of each evaluation index can be calculated as follows:

4 $$ {w_i} = \frac{{1 - {E_i}}}{{\sum\limits_{i = 1}^m {\left( {1 - {E_i}} \right)} }} $$

where $$ {w_j}$$ is the index weight and $$ \sum\limits_{j = 1}^n {{w_j} = 1} $$. The larger the entropy weight is, the greater the effect of the index on the scheme, in that it contains and transmits more decision information that has a greater influence on the final evaluation decision^39–44^.

### Combination weighting model based on game theory

This approach differs from the traditional simple linear combination weighting method. The central idea of this approach is to “coordinate conflicts and maximize benefits” by comprehensively considering the relationship between the indexes, balancing the subjective and objective weights, and optimising the index weight values. The basic algorithm is as follows:

### Construction of the basic weight vector set

Assuming that $$ H$$ weight values are obtained using the $$ H$$ weighting method, the basic weight vector set of the $$ H$$ method is

5 $$ {w_k} = \left( {{w_{k1}},{w_{k2}}, \cdots {w_{kn}}} \right),k = 1,2, \cdots ,H $$

Any linear combination of $$ H$$ weight vectors is

6 $$ w = \sum\limits_{k = 1}^H {{a_k}{w_k}^T} ,{a_k} > 0 $$

where $$ {a_k}$$ is the linear combination coefficient, and $$ w$$ is the comprehensive index weight value of the $$ H$$ weight set.

### Optimal combination weight

To find the balance between the different weights, the optimal effect weight vector $$ W$$ was obtained. In the calculation process, it is converted into an optimisation of the weight coefficient $$ {a_k}$$ to minimise the deviation between $$ w$$ and $$ {w_k}$$, as follows:

7 $$ min\left\| {\sum\limits_{j = 1}^H {{a_j}{W_j}^T - {W_i}^T} } \right\|,i = 1,2, \cdots ,H;j = 1,2 \cdots ,H $$

From the differential properties of the matrix, the first-order derivative condition for the optimisation of Eq. (7) becomes

8 $$ \sum\limits_{j = 1}^H {a_j} {W_i}W_j^T = {W_i}W_i^T $$

By solving Eq. (8), the combination coefficients $\left[ {{a_1},{a_2}, \cdots ,{a_H}} \right]$ can be obtained and normalised according to $a_k^* = {a_k}/\sum\limits_{k = 1}^H {a_k}$. The final combination index weight is $W = \sum\limits_{k = 1}^H {a_k^*W_k^T} ,k = 1,2, \cdots ,H$ ^31,32^.

### Workflow of extension matter element theory

The theoretical basis of extenics involves the matter element and extension set theories, and its logical cell is the matter element. As such, extenics introduces the concept of the matter element that organically combines quality and quantity. It is a triple group composed of things, features, and quantity values for things, which are depicted as R = (things, features, quantity values). The matter element concept correctly describes the relationship between quality and quantity, and it can be more appropriate to describe the change process of objective things. Different objects can have the same characteristic element and are represented by the matter element with the same characteristics. For convenience, many matter elements with the same characteristics are expressed in a simple way.

### Determination of the classical and joint domains

9 $$ {R_{ij}} = \left( {{N_j},{C_i},{V_{ij}}} \right) = \left[ {\begin{array}{*{20}{c}} {N_j}&{C_1}&{{V_{1j}}} \\ {}&{C_2}&{{V_{2j}}} \\ {}& \vdots & \vdots \\ {}&{C_i}&{{V_{ij}}} \end{array}} \right] = \left[ {\begin{array}{*{20}{c}} {N_j}&{C_1}&{\left( {{a_{1j}},{b_{1j}}} \right)} \\ {}&{C_2}&{\left( {{a_{2j}},{b_{2j}}} \right)} \\ {}& \vdots & \vdots \\ {}&{C_i}&{({a_{ij}},{b_{ij}})} \end{array}} \right] $$

Equation (9) is a matter element body with the same characteristics of a matter element with the same characteristics $$ {R_{ij}}$$, in which $$ {N_j}$$ is the $$ j$$ evaluation category, $$ {C_i}$$ is the $$ i$$ evaluation index, and $${V_{ij}} = \left( {{a_{ij}},{b_{ij}}} \right)\left( {i = 1,2, \cdots ,n;j = 1,2, \cdots ,m} \right)$$ is the range of quantity values $$ {N_j}$$ for the index $$ {C_i}$$, which is the classical domain of the data range taken by each category for the corresponding evaluation index.

10 $$ {R_P} = \left( {P,{C_i},{V_{iP}}} \right) = \left[ {\begin{array}{*{20}{c}} P&{C_1}&{{V_{1P}}} \\ {}&{C_2}&{{V_2}_P} \\ {}& \vdots & \vdots \\ {}&{C_n}&{{V_{nP}}} \end{array}} \right] = \left[ {\begin{array}{*{20}{c}} P&{C_1}&{\left( {{a_{1P}},{b_{1P}}} \right)} \\ {}&{C_2}&{\left( {{a_{2P}},{b_{2P}}} \right)} \\ {}& \vdots & \vdots \\ {}&{C_n}&{({a_{nP}},{b_{nP}})} \end{array}} \right] $$

where $$ P$$ is the whole of the category, $$ {V_{iP}}$$ is the range of quantity values taken of $$ P$$ for $$ {C_i}$$, and $$ {R_P}$$ is the $$ P$$ joint domain.

### Determination of the matter element to be evaluated

For $$ q$$ to be evaluated and using the matter element to express the detected data or analysis results, the matter element $$ {R_q}$$ to be evaluated can be expressed as

11 $$ {R_q} = \left( {q,{C_i},{v_i}} \right) = \left[ {\begin{array}{*{20}{c}} q&{C_1}&{v_1} \\ {}&{C_2}&{v_2} \\ {}& \vdots & \vdots \\ {}&{C_n}&{v_n} \end{array}} \right] $$

where $$ q$$ is some thing and $$ {v_i}$$ is the quantity value $$ q$$ for $$ {C_i}$$, which are the specific data obtained by the monitoring of the things that are to be evaluated.

### Determination and calculation of the degree of relation

Determination of the degree of relation for the thing to be evaluated in each category is expressed as follows:

$$ {K_j}\left( {v_i} \right) = = \left[ {\begin{array}{*{20}{c}} {\frac{{\rho \left( {{v_i},{V_{ij}}} \right)}}{{\rho \left( {{v_i},{V_{iP}}} \right) - \rho \left( {{v_i},{V_{ij}}} \right)}}\begin{array}{*{20}{c}} {}&{} \end{array}\rho \left( {{v_i},{V_{iP}}} \right) - \rho \left( {{v_i},{V_{ij}}} \right) \ne 0} \\ {\begin{array}{*{20}{c}} {}&{} \end{array} - \rho \left( {{v_i},{V_{ij}}} \right) - 1\begin{array}{*{20}{c}} {}&{}&{} \end{array}\rho \left( {{v_i},{V_{iP}}} \right) - \rho \left( {{v_i},{V_{ij}}} \right) = 0} \end{array}} \right] $$

where $$ \rho \left( {{v_i},{V_{ij}}} \right) = \rho \left( {{v_i},\left( {{a_{ij}},{b_{ij}}} \right)} \right) = \left| {{v_i} - \frac{{{a_{ij}} + {b_{ij}}}}{2}} \right|-\frac{{{b_{ij}} - {a_{ij}}}}{2}$$.

The calculation of the thing $$ q$$ to be evaluated for the degree of relation $$ j$$ is expressed as

$$ {K_j}\left( q \right) = \sum\limits_{i = 1}^n {{a_i}{K_j}\left( {v_i} \right)}  $$

### Determination of the level

Determination of the level is expressed as follows:

If $$ {K_{j0}} = \max \left\{ {K{}_j\left( q \right)} \right\},j \in \left( {1,2, \cdots ,m} \right)$$, $$ q$$ belongs to level $$ {j_0}$$.

In the extension set, the concept of a relational function is established. Any element in $$ U$$ can be quantitatively described by the relational function value, which can belong to the positive, negative, or zero domains (i.e., belongs to the elements in the same domain). It is also possible to separate different levels from the size of the relational function valu^27–30^.

## Result analysis and discussion

### Determination of the correlation function value for each evaluation index

Equation (12) allows the correlation function value of each evaluation index to be obtained (Table 3).

### Determination of index weights

According to the combination weighting method used in game theory, the combination weight formula is as follows:

14 $$ \left\{ {\begin{array}{*{20}{c}} {{a_1}{W_1}W_1^T + {a_2}{W_1}W_2^T = {W_1}W_1^T} \\ {{a_1}{W_2}W_1^T + {a_2}{W_2}W_2^T = {W_2}W_2^T} \end{array}} \right. $$

In this study, the weights of all indexes were calculated by AHP. First, according to the established risk evaluation index system of overseas mining investment, the importance of each index is determined by the expert grading method. To ensure the accuracy of the calculation results, 10 experts from universities, design institutes and multinational mining enterprises in the field of mining economic management were invited. According to the expert evaluation of different levels of indexes for different scales, the final evaluation results were calculated. Referring to the scaling table of AHP, the concrete values in the discrimination matrix can be obtained. Then, the index weight vectors reflecting each expert's opinions were calculated using the Maple software package layer by layer, and the consistency test was carried out.

Similarly, using the entropy weight method, the weights of all indexes were calculated according to Eqs. (1)-(4). Finally, the index weights obtained by the AHP and entropy method were combined, and optimal weight coefficients of $$ {a_1} = 0.9401$$ and $$ {a_2} = 0.1106$$ were obtained. These coefficients were then normalised, leading to $$ a_{_1}^{*} = 0.8894$$ and $$ a_{_2}^{*} = 0.1106$$. Using the expression $$ W = a_1^*W_{_1}^T + a_2^*W_{_2}^T$$, all the index weights were then finally calculated (Table 4).

**Table 4:** Calculated index weights.

_Index_	_AHP_	_Entropy weight method_	_Game theory_
Political stability	0.26	_0.07_	0.2422
Level of government corruption	0.04	_0.06_	0.0432
Sino-foreign friendship	0.04	_0.07_	0.0443
Mining policy	0.11	_0.05_	0.1030
Environmental standards	0.11	_0.05_	0.1030
Price level	0.15	_0.09_	0.1417
Economic growth rate	0.01	_0.06_	0.0195
Exchange rate	0.07	_0.09_	0.0711
Credit rating	0.03	_0.09_	0.0373
Humanistic environment	0.01	_0.07_	0.0129
Social security	0.04	_0.08_	0.0402
Trade union strike	0.01	_0.09_	0.0227
Information transmission	0.02	_0.07_	0.0252
Transportation	0.10	_0.06_	0.0937

### Data analysis of the index weights

**Figure 2:**
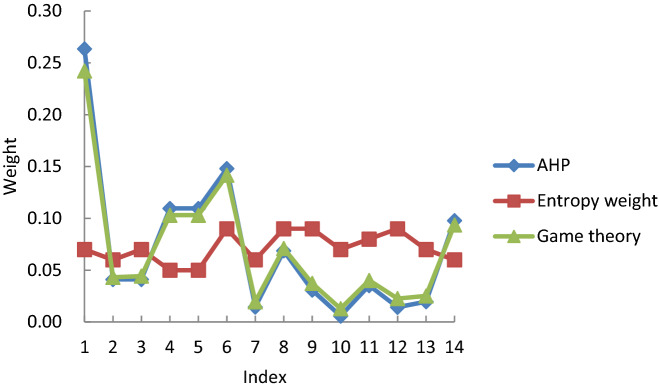
Index weights obtained from the three weighting methods.

Table 4 lists the results of a statistical analysis of the index weights calculated using the three different weighting methods. The distributions of the weights obtained by the three methods are also shown in Fig. 2. The index weights obtained by the AHP fluctuate significantly (Fig. 2) because this method is influenced by expert subjective factors that highlight the main factors but ignore the influence of some minor factors, thereby affecting the accuracy of the evaluation results. The index weights obtained by the entropy method are less variable because the method relies heavily on the original sample data, which are typically not very different. This results in relatively small differences in the index weight distribution, leading to inaccurate results. The index weights obtained by game theory are intermediate between those of the other two sets of values, and the weightings between the indexes are more balanced, as some minor factors and expert experience are better accounted for by game theory. As such, an optimal balance between subjective and objective factors is obtained with this approach.

### Determination of risk level

Equation (13) was used to calculate the relational degree of the environmental risk levels of mining investment in the Philippines (i.e.,${K_1}\left( q \right) = -0.85$,${K_2}\left( q \right) = -0.14$,${K_3}\left( q \right) = -0.58$,${K_4}\left( q \right) = -0.45$, and ${K_5}\left( q \right) = -0.64$). Accordingly, the environmental risk level of mining investment in the Philippines is level II (i.e., higher risk). This is higher than the risk level calculated by Zheng and Hu^8^ using the variable weight evaluation theory. However, our result is consistent with the current mining policy environment in the Philippines, which leads to a high-risk level for investing. Before companies undertake such investment, they must be familiar with the mining investment environment of the target resource country and consider possible types of investment risk in the future. The objective risks that cannot be avoided include political turmoil and social security, which can be assessed with our method and used to minimize potential economic losses caused by such risks.

## Conclusions

Scientific index weighting has an important influence on the environmental risk evaluation of overseas mining investment, which directly influences the accuracy of the results. In this paper, according to the limitations of the single weighting method presently used for the environmental risk evaluation of overseas mining investment, the subjective weight of each index was determined by the AHP, the objective weight was determined by the entropy weight method, and the overall weight was obtained using game theory. The final index weights take into account subjective and objective factors, including expert experience, and avoid the disadvantages of the single weight method.

A comprehensive evaluation of the environmental risks of overseas mining investment was constructed based on game theory and our extension matter element approach. The specific steps are as follows: first, the congener element object and classical domain of the environmental risk of mining investment are established based on extension matter element theory; then, the optimal index weights are obtained based on game theory, and the association function values of evaluation indexes and the environmental risk level of overseas mining investment are calculated; finally, the environmental risk level of overseas mining investment is determined based on the weight and association function values. The model was undertaken for the Philippines. The evaluation results show that the environmental risk level of mining investment in the Philippines was II (higher risk). According to the evaluation result of the environmental risk of mining investment in the Philippines, it is necessary to make prudent decisions for companies preparing to undertake overseas mining investments in the Philippines.

The extension matter element model based on game theory for evaluating the environmental risk of overseas mining investment yields robust and reliable results.
